# Schwannoma of the colon and rectum: a systematic literature review

**DOI:** 10.1186/s12957-018-1427-1

**Published:** 2018-07-03

**Authors:** Ali Bohlok, Melody El Khoury, Anne Bormans, Maria Gomez Galdon, Michael Vouche, Issam El Nakadi, Vincent Donckier, Gabriel Liberale

**Affiliations:** 1 Department of Surgical Oncology, Institut Jules Bordet, Université Libre de Bruxelles, Brussels, Belgium; 2 Institutional Library, Institut Jules Bordet, Université Libre de Bruxelles, Brussels, Belgium; 3 Department of Pathology, Institut Jules Bordet, Université Libre de Bruxelles, Brussels, Belgium; 4 Department of Radiology, Institut Jules Bordet, Université Libre de Bruxelles, Brussels, Belgium

**Keywords:** Schwannoma, Colon, Rectum, Review, Diagnosis, Treatment

## Abstract

**Background:**

Schwannomas of the colon and rectum are rare among gastrointestinal schwannomas. They are usually discovered incidentally as a submucosal mass on routine colonoscopy and diagnosed on pathologic examination of the operative specimen. Little information exists on the diagnosis and management of this rare entity.

The aim of this study is to report a case of cecal schwannoma and the results of a systematic review of colorectal schwannoma in the literature.

**Main body:**

PubMed, Scopus, and Cochrane database searches were performed for case reports and case series of colonic and rectal schwannoma.

Ninety-five patients with colonic or rectal schwannoma from 70 articles were included. Median age was 61.5 years (59% female). Presentation was asymptomatic (28%), rectorrhagia (23.2%), or abdominal pain (15.8%). Schwannoma occurred in the left and sigmoid colon in 36.8%, in the cecum and right colon in 30.5%, and in the rectum in 21.1%. Median tumor size was 3 cm and 56.2% of patients who underwent preoperative colonoscopy had a typical smooth submucosal mass. At pathology, 97.9, 13.7, and 5.3% of schwannomas stained positive for S100, vimentin, and GFAP, respectively. The median mitotic index was 1/50.

**Conclusions:**

Colorectal schwannoma is a very rare subtype of gastrointestinal schwannoma which occurs in the elderly, almost equally in men and women. Schwannoma should be included in the differential diagnosis of a submucosal lesion along with gastrointestinal stromal tumor, neuro-endocrine tumors, and leiomyoma-leiomyosarcoma. Definitive diagnosis is based on immunohistochemistry of the operative specimen. Rarely malignant, surgery is the mainstay of treatment.

## Background

Schwannomas of the gastrointestinal tract are spindle cell tumors originating from peripheral nerve lining Schwann cells and represent a very rare entity, accounting for approximately 2–6% of all mesenchymal tumors [1, 2]. In fact, the differential diagnosis of this entity includes all mesenchymal or neuro-ectodermal neoplasms, in decreasing frequency: gastrointestinal stromal tumors (GISTs), leiomyomas, leiomyosarcomas, neurofibromas, ganglioneuromas, paragangliomas, lipomas, granular cell tumors, and glomus tumors [3]. Gastrointestinal tract schwannomas occur in decreasing frequency in the stomach (83%), small bowel (12%), and, finally, the colon and rectum [1]. Gastrointestinal schwannomas occur at similar rates in men and women with a mean age of 60–65 years [3]. Most commonly, schwannoma is discovered incidentally on screening endoscopy or during abdominal imaging that is being done for another reason, and the diagnosis is made on definitive pathologic examination of operative specimen [4]. On immunohistology, they stain positive for S100. Gastrointestinal schwannomas occur indifferently in men and women with a mean age of 60–65 years [3]. Rectal schwannoma may cause symptoms such as obstruction, bleeding, and tenesmus. Few data exist on this rare entity in the literature.

The aim of this study is to report an incidental finding cecal schwannoma with a systematic literature review of colorectal schwannoma, to describe the clinical, diagnostic (endoscopic, abdominal imaging), pathologic, and prognostic features of colorectal schwannomas.

## Materials and methods

We performed a systematic review of the literature using three databases (PubMed, Scopus, and Cochrane). In the PubMed search, we used the following search terms: (“Colorectal Neoplasms”[Mesh]) AND “Neurilemmoma”[Mesh] with filter for case reports and (“Colorectal Neoplasms”[Mesh]) AND “Neurilemmoma”[Mesh] AND case series. Free terms included (colorectal OR rectum OR rectal OR colon OR colonic) AND Schwannoma and (colorectal OR rectum OR rectal OR colon OR colonic) AND Schwannoma AND case series. In the Scopus database search, we used the terms (TITLE-ABS-KEY ({case report})) AND (TITLE-ABS-KEY(schwannoma) AND TITLE-ABS-KEY(colorectal OR rectum OR rectal OR colon OR colonic)) OR ((TITLE-ABS-KEY(schwannoma) AND TITLE-ABS-KEY(colorectal OR rectum OR rectal OR colon OR colonic)) AND ({case series})). In the Cochrane search, we used the following: Title-abstract-keywords: Schwannoma and Title-abstract-keywords: Neurilemmoma. The search strategy had no publication date, or publication type restriction. In addition, the reference lists of relevant reviews or included articles were also searched to find other eligible studies. We included only studies published in English.

Study characteristics such as patient age, sex, and presenting symptoms, diagnostic exams, tumor characteristics (size, location, appearance on colonoscopy and on imaging), timing of diagnosis (endoscopic biopsy or post-operatively), type of resection (endoscopic, transanal, laparoscopic, and open), tumor immunostaining (S100, vimentin, glial fibrillary acidic protein [GFAP], Ki67), and mitotic rate reported in the pathology report, lymph node status, and the degree of aggressiveness of the tumor were evaluated in the literature review.

## Results

### Case report

**Fig. 1:**
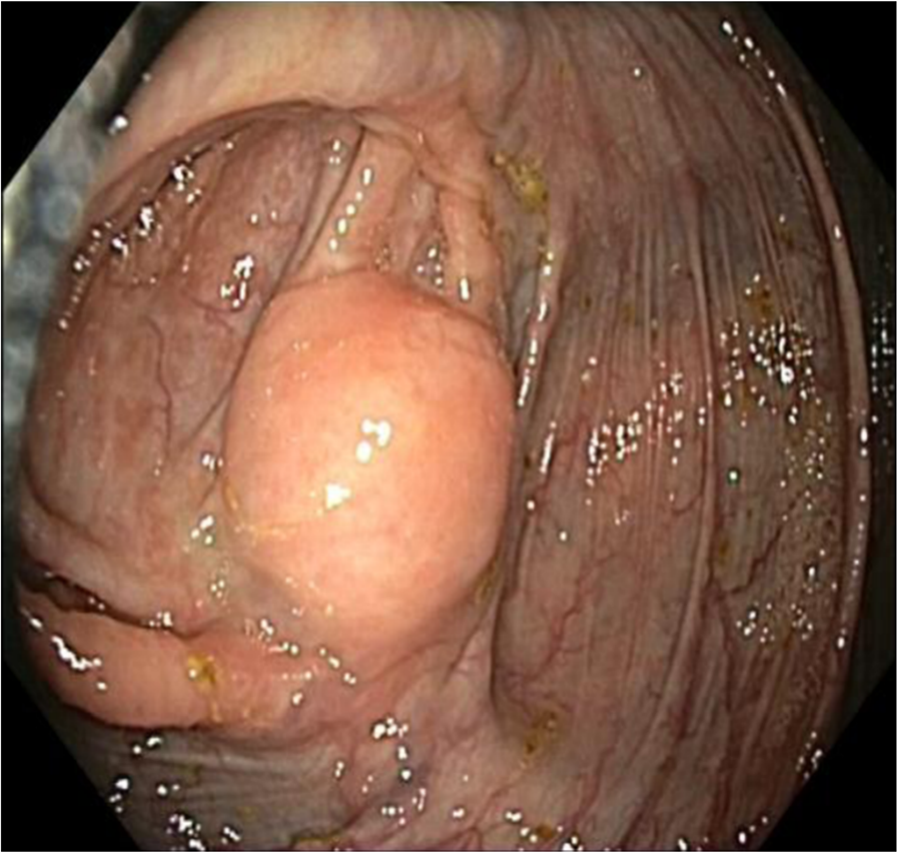
Colonoscopic view of the anterior submucosal mass

**Fig. 2:**
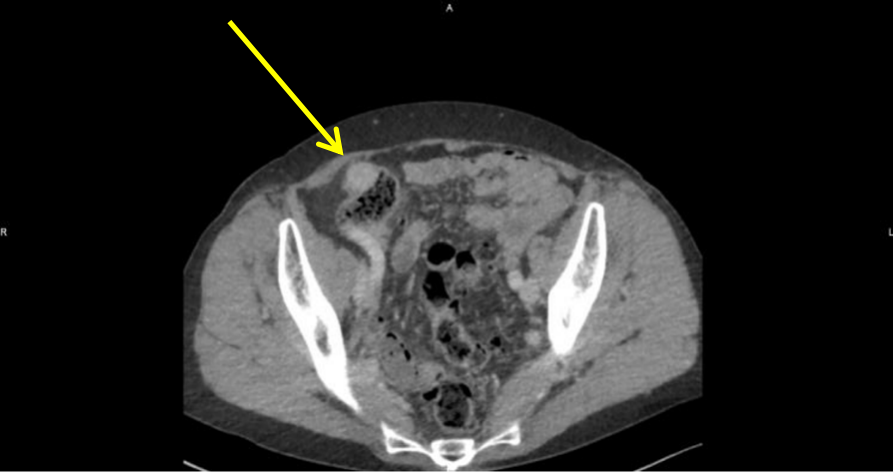
CT scan (axial) showing the hypervascular well-circumscribed cecal mass

**Fig. 3:**
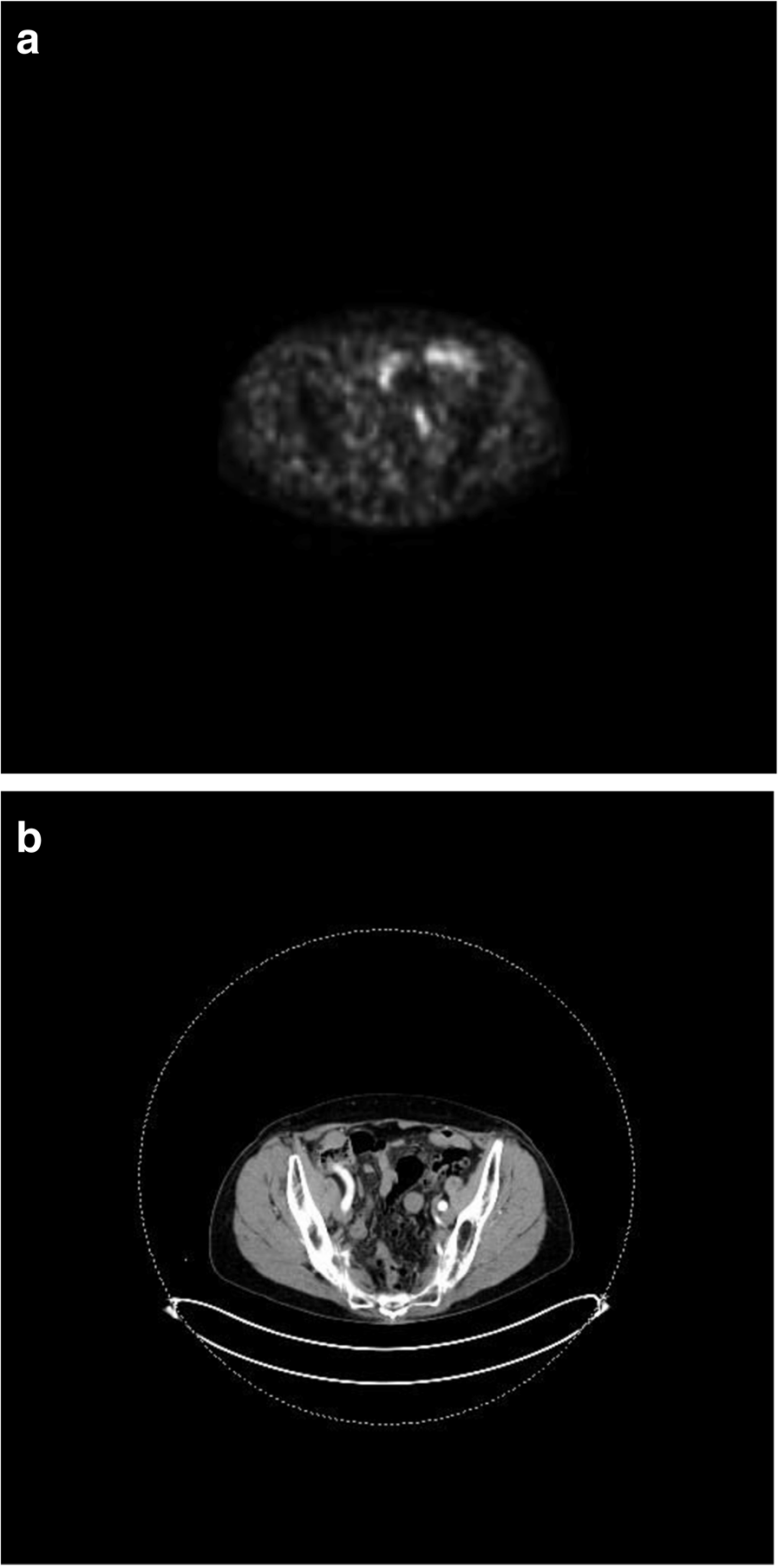
**a** Absence of metabolic activity on Octreo-PET/CT (axial). **b** Corresponding image on CT scan

An asymptomatic 70-year-old female known to have surgically treated squamous cell carcinoma of the nose underwent a routine screening colonoscopy that revealed an uncomplicated diverticulosis and a cecal submucosal mass (Fig. 1). The appearance was most likely correlated with a submucosal tumor and less likely to be an extrinsic compression. At pathology, colonoscopic biopsies of the mass showed normal colonic mucosa. Laboratory examination showed no anemia (hb 13.2 g/dL) was negative for CEA tumor marker (CEA 2.2 μg/L). Abdominal computed tomography scan (CT scan) revealed a well-circumscribed hypervascular anterior cecal wall mass (Fig. 2) with no liver metastases and no other distant lesions. The mass had no metabolic activity on either FDG-PET scan or on Octreo-PET (Fig. 3a, b).

**Fig. 4:**
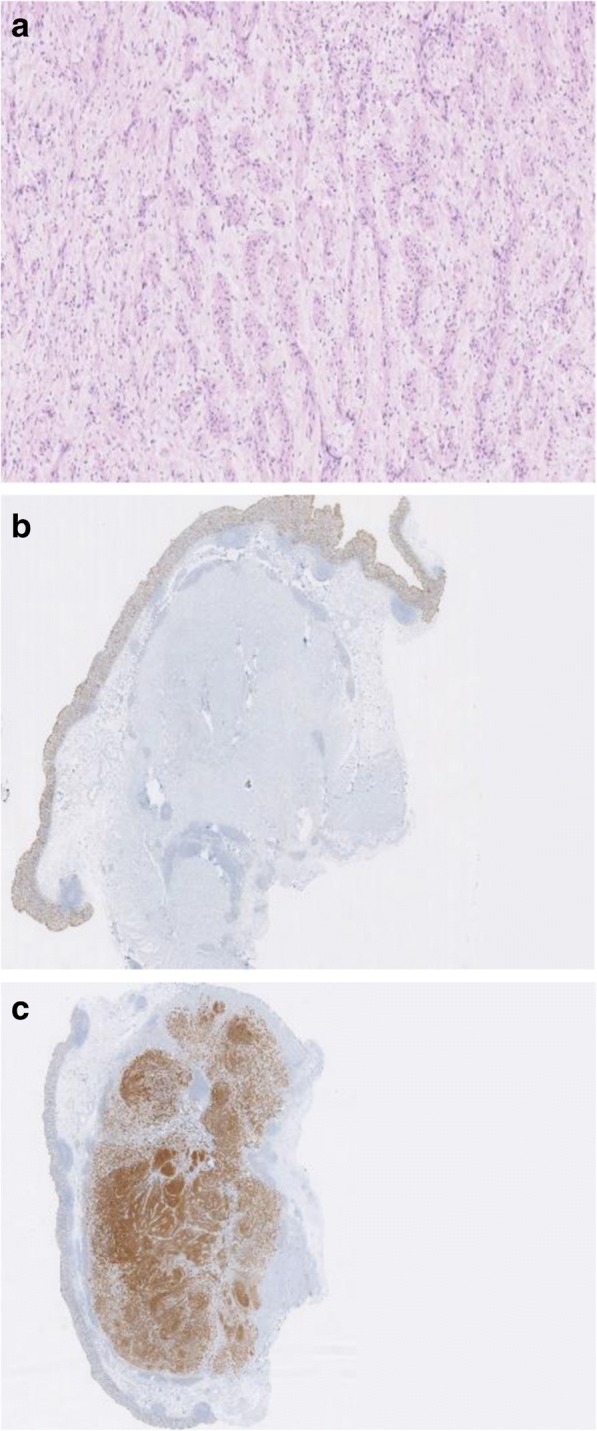
**a** Hematoxylin eosin (HE) staining showing the spindle cell type tumor. **b** Absence of tumor immunohistochemistry (IHC) staining for CD117. **c** Tumor IHC staining for S100

After multidisciplinary team discussion, a differential diagnosis of mesenchymal tumor of the colon (GIST, leiomyoma, and leiomyosarcoma) was suggested and we decided to perform an exploratory surgery. The patient was consented for open exploration by mini-laparotomy and possible right hemi-colectomy. The right colon was mobilized at the white line of Toldt, the 3 cm white cecal mass was well circumscribed, and a wedge resection, including the appendix, using GIA 75 (Ethicon Endo-Surgery GIA; 75 mm; Guaynabo, Puerto Rico 00969 USA) was performed. The operative specimen was sent for frozen section at pathology. The temporary diagnosis was a benign spindle cell tumor. The intra-operative decision was to wait for the definitive histopathologic examination report in order to try to avoid a right hemicolectomy. The final pathology report revealed a benign spindle cell tumor that stained negative for CD117 and DOG-1 and was diagnosed as cecal schwannoma with a reactive lymph node (Fig. 4).

The post-operative course was uneventful and the patient started oral feeding the same night and was discharged on day 5 with pain killers. The final multidisciplinary committee decision was follow-up without further treatment needed. At 1-year follow-up, the patient is disease free.

### Systematic literature review

#### Study selection

**Fig. 5:**
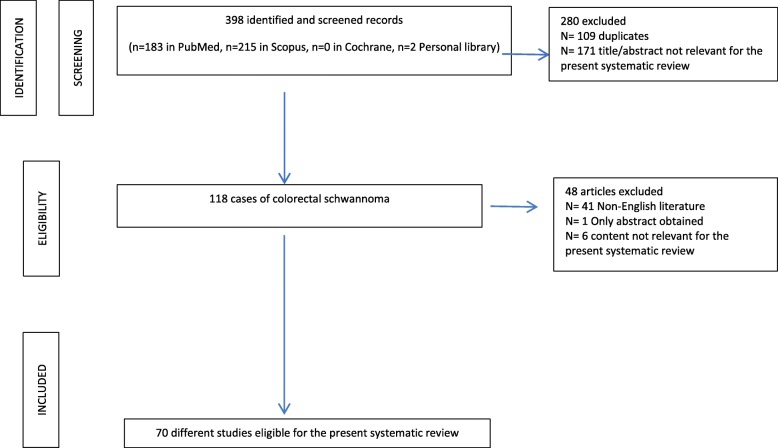
PRISMA flowchart summarizing the process for the identification of eligible studies

A total of 521 articles were identified from the PubMed, Scopus, and Cochrane databases. After removing duplicated articles, 230 articles remained for further assessment. A total of 171 articles were excluded on the basis of the titles and the abstracts. Of the remaining 120 articles, only 78 articles had full text published in English. After full text review of these remaining articles, 70 were eligible and included in the systematic review (Fig. 5).

#### Patient and tumor characteristics

A total of 70 articles (Table 1) reporting 95 cases of colorectal schwannoma were found, including one article reporting a series of 20 colorectal schwannomas from the Armed Forces Institute of Pathology [4]. A total of 96 patients were reviewed, including our case [1, 2, 4–71]. The statistical analysis of all patient characteristics is listed in Table 2.

**Table 1:** Schwannomas of the colon and rectum: clinical and histopathologic characteristics of published case reports

Study	Age	G	Symptoms	*S*	Location	Colonoscopy and imaging	Diagnosis	Surgery/LN	(Antonini) S-100 +/mitosis
Tsunoda [1]	67	F	FOB	3	Transverse	Ulcerated S-M/ CT: well circumscribed, homogenous enhancement, EUS: hypo-echoic S-M	Post-Sur	Open/−	NSE/MIB-1 low
Nonose [2]	71	F	Tenesmus	3	Sigmoid	Ulcerated S-polyp	Biopsy	Lap/−	(A)Ki67 < 5%
Miettinem [4] 20	82	M	NA	0.5	Rectum	S-M/NA	Biopsy	Endoscopy/NA	Mitosis 0/50
	30	F	FOB	1.5	Sigmoid	S-M/NA	Biopsy	Endoscopy/NA	Mitosis 2/50
	83	M	NA	2.3	Cecum	NA	Post-Sur	Open/NA	Mitosis 1/50
	64	F	Asymptomatic	2.5	Transverse	NA	Post-Sur	Open/NA	Mitosis 1/50
	62	M	Rectorrhagia	2.5	Left	NA	Post-Sur	Open/NA	Mitosis 0/50
	65	M	Asymptomatic	2.8	Cecum	Cecal mass/NA	Post-Sur	Open/NA	Mitosis 0/50
	80	F	NA	3	Sigmoid	S-polyp/NA	Post-Sur	Open/NA	Mitosis 1/50
	71	F	Rectorrhagia	3	Cecum	NA	Post-Sur	Open/NA	Mitosis 0/50
	58	M	Rectorrhagia	3	Transverse	NA	Biopsy	Open/NA	Mitosis 0/50
	57	F	Rectorrhagia	3	Sigmoid	S-M/NA	Post-Sur	Open/NA	Mitosis 1/50
	84	F	Asymptomatic	3.9	Cecum	NA	Biopsy	Open^b^/NA	Mitosis 0/50
	87	M	NA	4	NA	NA	Post-Sur	NA	Mitosis 5/50
	75	F	Constipation	4	Transverse	S-M/NA	Post-Sur	Open/NA	Mitosis 1/50
	18	F	Intussusception	5	Cecum	S-M/NA	Post-Sur	Open/NA	Mitosis 3/50
	72	M	NA	5.5	Cecum	S-M/NA	Post-Sur	Open/NA	Mitosis 0/50
	55	M	NA	0.7	Sigmoid	S-M/NA	Post-Sur	Open/NA	Mitosis 0/50
	58	F	NA	0.8	Sigmoid	NA	Post-Sur	Open/NA	Mitosis 0/50
	72	M	Weight loss^a^	1	Sigmoid	S-polyp/NA	Biopsy	Endoscopy/NA	Mitosis 0/50
	61	F	Ulcer symptoms	5	Sigmoid	NA	Post-Sur	Open/NA	Mitosis 0/50
	23	F	Asymptomatic	5.5	Sigmoid	NA	Post-Sur	Open/NA	Mitosis 1/50
Barbeiro [5]	49	F	Asymptomatic	3	Right	S-M/CT: well circumscribed, homogenous enhancement	Post-Sur	Open/−	Low mitosis
Jung [6]	59	F	Rectorrhagia	4.8	Sigmoid	Fungating mass/CT: enlarged LN, FDG-PET: Hyper	Post-Sur	Open/−	Mitosis < 1/10
Park [7] 2	52	F	NA	1.3	Right	S-M/CT: well circumscribed, homogenous enhancement	Post-Sur	Lap/NA	NA
	59	F	Asymptomatic	1.5	Left	S-M/CT: well circumscribed	Post-Sur	Lap/NA	NA
Hornick [8]	48	M	NA	5	NA	NA	NA	NA	NA
Akgul [9]	57	F	Tenesmus	6.5	Rectum	S-M/ CT: isodense + central necrosis	Post-Sur	Open/NA	NA
Wang [10]	62	F	Rectorrhagia^a^	4	Right	S-polyp/CT: well circumscribed, homogenous low enhancement	Post-Sur	Open/NA	(A) Ki-67 < 3%
Tashiro [11]	64	F	Asymptomatic	5	Right	S-M/CT: well circumscribed, homogenous enhancement	Post-Sur	Lap/−	MIB-1 < 5%
Suzuki [12]	70	F	Asymptomatic	18	Rectum	S-M/ MRI: hypo T1- hyper T2, EUS: hypo-echoic S-M	Post-Sur	Transanal μ/NA	< 1/10
Bugiantella [13]	65	M	FOB	2.5	Sigmoid	S-M/ EUS: hypo-echoic S-M	Post-Sur	Lap/−	Vimentin/Ki-67 < 2%
De Mesquita [14]	79	F	Weight loss^a^	10	Cecum	NA/CT: well circumscribed, homogenous low enhancement	Post-Sur	Open/NA	Vimentin/NA
Vasilakaki [15]	68	M	Rectorrhagia	4	Right	S-M/NA	Post-Sur	Open/−	NA
Kanneganti [16]	35	F	Melena^a^	4	Cecum	Ulcerated S-M/CT: mass	Post-Sur	Open/NA	low
Goh [17] 2	54	F	Abdominal pain	3	Right	Obstructing mass/NA	Post-Sur	Open/NA	NA
	41	F	Rectorrhagia	2.9	Rectum	S-M/EUS: S-M	Post-Sur	Transanal/NA	NA
Inagawa [18] 2	73	F	Rectorrhagia^a^	3.5	Sigmoid	Hard ulcerated S-M/CT: high density	Post-Sur	Open/NA	(A)vimentin, GFAP/NA
	44	M	FOB	1	Cecum	S-M/NA	Biopsy	Endoscopy/NA	(A) vimentin, GFAP/NA
Turaihi [19]	61	F	Rectorrhagia	4	Cecum	S-M/CT: mass	Post-Sur	Lap/−	NA
Uhr [20]	73	F	Asymptomatic	3.1	Left	S-M/CT: mass + enlarged LN	Post-Sur	Lap/NA	(A)NA
Meeks [21]	95	F	Obstruction	1	Appendix	NA	Post-Sur	Open/NA	NA
Terada [22]	52	M	Asymptomatic	0.9	NA	S-M	Biopsy	Endoscopy/NA	NA
Çakir [23]	79	F	Rectorrhagia	5	Sigmoid	Rough ulcerated mass/CT: ulcerated mass	Post-Sur	Open/−	Vimentin/Ki-67 < 5%
Dickson [24]	72	M	Rectorrhagia^a^		Cecum	S-M/CT: mass	Post-Sur	Open/NA	(A, B) NA
Tokuhara [25]	74	F	FOB	3	Sigmoid	S-M/EUS: hypo-echoic S-M	Post-Sur	Lap/NA	(A)MIB-1 < 3%
Baskaran [26]	49	M	FOB		Pancolic	S-M/NA	Biopsy	Endoscopy/NA	NA
Verdu-Fernandez [27]	67	M	Asymptomatic	4.5	Left	NA/CT: mass with enlarged LN, US: intussusception	Post-Sur	Open/NA	CD68
Baek [28]	70	F	Asymptomatic	2	Cecum	Polyp/CT: polyp with low attenuation	Post-Sur	Lap/NA	(A) CD34/ Ki-67 < 5%
Petrie [29]	25	M	Bloody diarrhea^a^	4	Sigmoid	Friable mucosa/CT: obstructing mass	Post-Sur	Open/NA	(A)NA
Shelat [30]	63	F	Anorexia^a^		Rectum	S-polyp/CT: polyp	Post-Sur	Open/−	NA
Trivedi [31]	61	M	NA	1.3	Sigmoid	S-polyp/NA	Post-Sur	Endoscopy/NA	CD34/MIB-1 < 2%
Kawaguchi [32]	77	F	Tenesmus	1	Rectum	S-M/EUS: hypoechoic, CT: mass	Biopsy	Endoscopy/NA	(A) mitosis < 1/10
Tan [33]	63	F	Early satiety	3	Rectum	S-polyp/MRI: mass	Post-Sur	Open/ NA	NA
Yang [34] 2	27	F	Rectorrhagia	3	Rectum	S-M/NA	Post-Sur	NA	NA
	53	M	Asymptomatic	2	Sigmoid	S-M/NA	Post-Sur	NA	NA
Matsumoto [35]	59	F	Asymptomatic	2	Cecum	S-M/CT: well circumscribed, homogenous enhancement	Post-Sur	Lap wedge/NA	Vimentin/ MIB-1 < 2%
Kim [36]	61	M	Asymptomatic	1.8	Right	Polypoid/CT: well circumscribed, homogenous enhancement	Biopsy	Lap/NA	NA
Vijayasekaran [37]	75	F	Asymptomatic	3.2	Rectum	NA/EUS: S-M, CT: enlarged LN	Post-Sur	Open	NA
Wu [38]	84	M	NA		Rectum	NA: FDG PET: hyper	Post-Sur	NA	NA
Tedeschi [39]	80	M	Rectorragia	4	Rectum	S-polyp/CT: mass	Post-Sur	Open/NA	(A)mitosis < 1/10
Tanaka [40]	70	F	FOB		Left	Ulcerated S-M/CT: well circumscribed, homogenous enhancement	Post-Sur	Open/NA	(A,B)NA
Rocco [41]	67	F	Asymptomatic	0.3	Left	S-polyp/NA	Biopsy	Endoscopy/NA	NA
Wani [42]	75	F	Abdominal pain	5	Right	S-polyp/NA	Post-Sur	Open/NA	NA
Kienemud [43] 2	70	F	Asymptomatic	0.7	Sigmoid	S-M/NA	Post-Sur	Open/NA	(B)NA
	70	M	Asymptomatic	1.3	Sigmoid	S-polyp/NA	Biopsy	Endoscopy/NA	(B)NA
Hsu KF [44]	88	F	Constipation	3.5	Rectum	S-M/EUS: hypoechoic, CT: no enlarged LN	Post-Sur	Transanal/NA	(A,B)NA
Wilde [45]	68	F	Explosive diarrhea,	1.5	Sigmoid	NA	Post-Sur	Open/−	(B)vimentin, GFAP/NA
Mysorekar [46]	33	M	Abdominal pain	3.5	Right	S-polyp	Post-Sur	Endoscopy/NA	(A,B)NA
Lee [47]	32	F	Constipation	1.4	Right	S-polyp	Biopsy	Endoscopy/NA	NA
Chetty [48]	43	F	Asymptomatic		NA	S-polyp	Biopsy	Endoscopy/NA	HMB-45, Melan-A/NA
Brauman [49]	55	M	Constipation	5	Right	S-polyp/CT: mass with enlarged LN	Post-Sur	Open/NA	Β-catenin, CD117/MIB-1 < 5%
Zippi [50]	72	M	Abdominal pain	1.5	Rectum	S-M/NA	Biopsy	Endoscopy/NA	(A,B) NA
Hsu CT [51]	68	M	Abdominal pain	3.5	Left	S-M/ CT: well circumscribed, low homogenous enhancement,	Post-Sur	Open/NA	NA
Emanuel [52]	48	M	Rectorrhagia	4.9	Left	Obstructing mass/CT: intussusception	Post-Sur	Open/NA	(A)EMA, CD34, vimentin/NA
Fotiadis [53]	55	M	Rectorrhagia		Sigmoid	S-M/CT: no metastasis	Post-Sur	Open/NA	(B)NA
Bhardwaj [54]	35	F	Tenesmus	2.3	Rectum	Mass/NA	Post-Sur	Open/NA	(A)GFAP/NA
Jacobson [55]	56	F	Asymptomatic	1.7	Sigmoid	S-M/NA	Post-Sur	Open/NA	(A)mitosis < 1/10
Maciejewski [56]	67	F	Abdominal pain	8	Rectum	S-M/US: large exophytic mass	Post-Sur	Open wedge/NA	(A)vimentin/NA
Matsushita [57]	79	M	NA		Transverse	S-M	Biopsy	Endoscopy/NA	NA
Horio [58]	66	M	FOB	3	Sigmoid	S-M	Post-Sur	Open/NA	Vimentin/NA
Sasatomi [59]	68	F	Rectorrhagia	4.7	Sigmoid	S-M/CT: mass	Post-Sur	Open/−	NA
Prévot [60]	74	M	NA	2	Right	NA	Post-Sur	Open/NA	Vimentin, NSE/NA
Tomozawa [61]	66	M	Asymptomatic	3.5	Right	Polypoid mass/CT: no enlarged LN	Biopsy	Open/NA	(A,B)vimentin/mitosis < 1/80
Skopelitou [62]	69	F	Rectorrhagia	3.5	Left	Polypoid mass/barium enema: obstruction	**NA**	Open/NA	GFAP, leu7/mitosis < 3/10
Kakizoe [63]	72	M	Asymptomatic	1.5	Rectum	S-M/NA	Post-Sur	Transanal μ/NA	(A)NA
Murakami [64]	75	M	Asymptomatic	2.2	Rectum	Ulcerated S-M/NA	Biopsy	Transanal/NA	NA
Sugimura [65]	14	M	Melena		Sigmoid	NA/barium enema: mass, CT: mass of soft tissue	Post-Sur	Open/NA	(A)vimentin/NA
Abe [66] 2	52	M	Protruding mass	1.5	Rectum	S-M	Biopsy	Transanal/NA	(A)NA
	52	F	Rectal discomfort	4.5	Rectum	Polyp	Biopsy	Transanal/NA	(B)NA
Schwartz [67]	18	F	↗ Abdominal girth	28	Sigmoid	NA	Post-Sur	Open/NA	(A)NA (malignant)
Cleveland [68]	60	F	↗ Abdominal girth	20	Transverse	NA/abdominal X-ray: large mass aortogram	Post-Sur	Open/NA	NA
Bodner [69]	63	F	Constipation		Rectum	Recurrence, malignant transformation	Biopsy	Abdominal	Malignant
Wang CL [70]	77	F	NA	2.4	Cecum	NA/CT: well circumscribed, low homogenous enhancement FDG PET: hyper	Biopsy	Open/NA	(A,B)NA
Catania [71]	41	M	Tenesmus	8	Rectum	NA/heterogenous large mass	Post-surg	Open/−	c-Kit/NA Malignant
Present case	70	F	Asymptomatic	2	Cecum	S-M/CT: well circumscribed, homogenous enhancement, FDG PET: hypo, Octreo PET: hypo	Post-Sur	Openwedge/−	< 1/10

*S-M* submucosal mass, *S-polyp* submucosal polyp, *NA* not available, − negative

^a^Associated with abdominal pain

^b^Enucleation

**Table 2:** Patient characteristics

Characteristics	
Number	95
Age	61.2 years, range 14–95, median 64 years
Sex	
Male	40 (41.7%)
Female	56 (58.3%)
Presenting symptom	
Asymptomatic	27 (28.1%)
Rectal bleeding	22 (22.9%)
Fecal occult blood	8 (8.3%)
Abdominal pain	15 (15.6%)
Constipation	7 (7.3%)
Tenesmus	7 (7.3%)
↗ Abdominal girth	2 (2.1%)
NA	14 (14.6%)
Location	
Cecum and right colon	29 (30.2%)
Transverse colon	5 (5.2%)
Sigmoid	27 (28.1%)
Left colon	8 (8.3%)
Rectum	21 (21.9%)
Appendix	1 (1%)
NS	5 (5.2%)
Colonoscopy	73 (76%)
Smooth submucosal mass	41 (56.2%)
Ulcerated submucosal mass	5 (6.8%)
Submucosal polyp	15 (20.5%)
Intraluminal mass	6 (8.2%)
Polypoid	3 (4.1%)
Others	3 (4.1%)
Imaging	46 (48%)
CT-SCAN	35 (76%)
Well circumscribed	13 (37.1%)
Homogenous enhancement	12 (34.3%)
Central necrosis	1 (1%)
EUS: hypo-echoic mass	7 (7.3%)
FDG-PET	4 (4.2%)
Hyper	3 (3.1%)
Size (cm)	3.78 cm, range 0.3–28, median 3 cm
Diagnosis	
Endoscopy	23 (24%)
Post-surgery	71 (74%)
Type of surgery	
Laparoscopic	11 (11.5%)
Open	58 (60.4%)
Endoscopic	15 (15.6%)
Transanal	7 (7.3%)
NA	5 (5.2%)
Extent of surgery	
Classical segmental	65 (67.7%)
Wedge	3 (3.1%)
Endo-luminal	23 (24%)
Tumor markers	
S100	94 (97.9%)
Vimentin	13 (13.5%)
GFAP	5 (5.2%)
CD-34	2 (2.1%)
CD-68	1 (1%)
Antonini type	26 (27.1%)
A	15 (57.7%)
B	6 (23.1%)
A, B	5 (19.2%)
Mitosis	
Ki-67%	5 (5.2%)
MIB-1	6 (6.3%)
Low mitosis	30 (31.3%), mean 2.1/50, median 1/50
NA	55 (57.3%)
LN	
NA	85 (88.5%)
Negative	11 (11.5%)
Aggressiveness	
Benign	93 (96.9%)
Malignant	3 (3.1%)

There were 40 male (41.7%) and 56 female (58.3%) patients with a mean age of 61.2 years (range 14 to 95 years). Thirty-five patients (36.4%) were asymptomatic at presentation, including eight with positive fecal occult blood test (8.3%). The most common presenting symptoms were rectorrhagia (22.9%) followed by nonspecific abdominal pain (15.6%), constipation (7.3%), tenesmus (7.3%), and increased abdominal girth (2.1%).

Schwannoma occurred most frequently in the cecum and right colon (30.5%) followed by the sigmoid (28.1%), the rectum (21.1%), the left colon (8.3%), the transverse colon (5.3%), and the appendix (1.1%). The tumor size ranged from 0.3 to 28 cm with a mean of 3.78 cm (median 3 cm).

On colonoscopy, results were available in 73 out of 96 patients (76%). Schwannoma had the typical submucosal mass appearance with smooth mucosal surface in 41 (56.2%) and with ulcerated mucosa in 5 (6.8%). Fifteen patients (20.5%) had a tumor described as submucosal polyp, and nine patient tumors (12.3%) were described as a mass, either fungating (8.2%) or polypoid (4.1%).

Pre-operative tumor imaging results were available for 46 patients (48%). The most common imaging done was abdominal CT scan (35 patients, 76%). In the majority, the CT scan report lacked a description of the schwannoma and identified a colorectal mass. In 12 cases (34.3%), the schwannoma was described as a well-circumscribed homogenous lesion with low enhancement on arterial phase and in one case as a well-circumscribed homogenous lesion without arterial phase enhancement. Endorectal ultrasound (EUS) results were available for only seven patients (7.3%) and all of these showed a transmural hypo-echogenic mass. FDG-PET/CT scan was done in only four patients (including our patient) and showed a hypermetabolic lesion in three of them.

The diagnosis of schwannoma was made on the operative specimen in the majority of patients (74%), on endoscopic or transanal biopsy in 23 patients (24%), and diagnostic method was not reported in 2% of cases.

All patients with no biopsy or inconclusive biopsies underwent radical colonic resection either open (60.4%) or laparoscopic (11.5%). Three patients were diagnosed with schwannoma pre-operatively and underwent wedge colonic resection. Fifteen patients had colonoscopic resection at their initial examination and resection was judged sufficient and they did not undergo further treatment. Transanal surgery was performed in seven patients with rectal schwannomas including two treated by transanal microsurgery.

On pathologic examination, the Antonini subtype was available or deduced in 26 out of 96 patients, 57.7% were type A (14 patients), half of the remaining were type B, and the other half were both types A and B. In all available immuno-histologic examinations, schwannomas stained positive for S100 in 97.9%, for vimentin in 13.5%, for glial fibrillary acidic protein (GFAP) in 5.2%, for CD34 in 2.1%, and for CD68 in 1%. Tumor mitotic activity results were reported in only 41 patients (Ki-67 in 5 patients (5.3%) the highest below 5%, MIB-1 in 6 patients (6.3%) the highest < 5%, and low mitotic count in 30 (31.6%) with a mean of 2.1/50 and a median of 1/50 *high-power fields*). Lymph node status was available in only 11 pathology reports (11.5%) and was negative in all of these. Schwannoma was judged to be benign in 93 out 95 patients (96.9%), and local and hepatic pattern recurrences were observed in 3 patients (3.1%) and were reported as “malignant” schwannomas [67, 69, 71].

## Discussion

Schwannomas are extremely rare tumors of the nerve sheath, developing from Schwann cells. In the gastrointestinal tract, they present as spindle cell tumors, originating from Auerbach’s myenteric plexus rather than Meissner’s submucosal plexus, and account for approximately 2–6% of all mesenchymal tumors [1].

Colorectal schwannoma is a very rare neoplasm and is the least frequent location for a GI schwannoma [1, 2]. Based on our systematic review, colorectal schwannoma occurs slightly more in female patients (59%), with a mean age of 61.5 years, and a wide age range from 14 to 95 years. Schwannoma is frequently diagnosed as a submucosal mass or polyp [4, 10, 28, 30, 31, 33, 39, 41–43, 46–49, 66] with a smooth surface but in rare cases can ulcerate into the mucosa [1, 2, 16, 18, 23, 29, 40]. This submucosal mass is usually discovered incidentally during routine screening colonoscopy. As is true for all other mesenchymal tumors, mucosal biopsy is usually inconclusive. Deep biopsy or submucosal resection [4, 18, 22, 26, 31, 41, 43, 46–48, 50, 57] can help differentiate schwannoma from other mesenchymal tumors such as gastrointestinal stromal tumors (GIST), neuro-endocrine tumors (NET), leiomyomas, and leiomyoma-sarcomas, or from adenocarcinomas in cases where the mucosa is ulcerated. In decreasing frequency, schwannoma occurs in the right colon and cecum, followed by the sigmoid colon, the rectum, the left colon, and, finally, the transverse colon. The size of schwannomas ranges from less than 1 cm lesions to very large tumors up to 28 cm that present with an increase in abdominal girth [67, 68].

For differential diagnosis of schwannoma, abdominal CT scan can help differentiate between schwannoma and other mesenchymal tumors. Schwannomas appear as well-defined homogenous mural masses with low enhancement [1, 5, 7, 10, 11, 14, 35, 36, 40, 51, 70] in comparison with the heterogenous aspect of GIST and the ill-defined aspect of adenocarcinomas [72]. Less than half of the published case reports had a CT scan done, approximately half of these had endoluminal resection and did not require abdominal imaging. In two thirds of patients who underwent CT scan, the published case reports lacked a description of the colorectal mass scan and did not specify the characteristics of the mass with regard to shape and arterial enhancement, factors which might be helpful for differential diagnosis. Echo-endoscopic ultrasonography can also be useful for diagnosis as schwannoma appears as a well-defined transmural hypoechoic mass.

In this review, four patients (4.16%) underwent metabolic imaging (FDG-PET/CT scan). Schwannoma exhibited hypermetabolic activity in three of these (75%) and no metabolic activity in one of them (25%). In the majority of cases, FDG-PET/CT scan was performed in the preoperative work-up of an atypical colorectal mass to differentiate between a malignant and a benign lesion, as in our case report. In patients with and without metabolic activity, all lesions were reported as benign. Although reported data are limited, current data do not suggest a role for FDG-PET/CT scan to differentiate between benign and malignant gastrointestinal schwannoma. In addition, octreotide receptor PET/CT scan can help to exclude the diagnosis of NET, as in the present report.

The definitive diagnosis is made on immunohistopathologic examination of the operative specimen. Macroscopically, schwannomas tend to be lobulated well-defined tumors ulcerating into the mucosa [73]. Furthermore, they stain positive for S100, and occasionally for vimentin, and stain negative for SMA, Desmin, CD117, and P53 [74]. One of the malignant schwannomas [71] had a c-kit mutation along with S100 which makes a diagnosis of GIST more likely, although the tumor was considered to be a malignant schwannoma.

Two histological growth patterns have been described: Antoni A, characterized by the dense growth of fusiform cells compactly arranged in palisades to form Verocay bodies and Antoni B in which the fusiform cells are more loosely distributed with rounded or elongated nuclei, with a great quantity of myxoid stroma and xanthomatous histiocytes. Recognition of these patterns has proved useful in the histologic identification of schwannomas [2].

Colorectal schwannomas are reported as benign in more than 98% of cases. They are characterized by a low rate of mitosis, the absence of atypical mitotic figures, and nuclear hyperpigmentation. The degree of aggressiveness depends on the Ki-67 index and the mitotic index. Ki-67 index is recommended as an indicator of malignancy. A value of more than 5% correlates with greater tumor aggressiveness and a value of more than 10% is considered malignant [2]. A higher risk of metastasis and/or recurrence has been associated with a mitotic activity rate > 5 mitoses per field at high magnification and a tumor size larger than 5 cm [8]. More than half of the published case reports lacked the complete pathologic description of the schwannoma, and the differentiation between benign and malignant was not based on mitotic index, Ki-67, or MIB-1. Malignant profile was judged based on long-term local and distal recurrence [67, 69, 71]. Metastatic spreading in lymph nodes is exceptional but Das Gupta and Brasfield have reported the occurrence of loco-regional metastases in aggressive tumors (2%) [9]. Three cases of malignant colorectal schwannoma were reported [67, 69, 71] but they lacked data on mitotic activity, Ki-67, and MIB-1. In these case reports, the authors made their conclusion depending on the large size with numerous mitoses [67] and on the emergence of long-term local recurrence and liver metastasis [69–71].

The best therapeutic option is complete surgical resection with free negative margins. Radical surgery is not necessary. In reported cases, the observed high frequency of radical resection is due to the absence of accurate preoperative diagnosis. When diagnosed preoperatively, schwannomas were resected either endoscopically or by a wedge resection [35, 56]. According to our research, no patients were offered adjuvant radiotherapy or chemotherapy.

Our review was limited by the small number of published case reports and the low index of pre-operative suspicion which resulted in somewhat hazardous diagnostic examinations. Most patients either did not undergo an abdominal imaging modality or lacked a detailed description of imaging results. Moreover, mitotic index was not calculated in all patients.

## Conclusions

In conclusion, schwannoma of the gastrointestinal tract is a rare, usually benign, tumor, and colonic schwannoma is an even more rare occurrence. Differential diagnosis of a submucosal lesion should include schwannoma as well as GIST, NET, and leiomyoma-leiomyosarcoma. Submucosal or deep biopsy might help to make a pre-operative diagnosis. Contrast-enhanced CT scan with low enhancement could help differentiate the well-defined schwannoma from other mesenchymal tumors or adenocarcinoma and exclude distant metastasis in cases of other diagnoses. The definitive diagnosis is based on immunohistochemistry of the operative specimen. Schwannoma stains strongly for S100, and the mitotic index should be calculated to help differentiate benign from malignant lesions. Surgery is the mainstay of treatment, and as for other mesenchymal tumors, wedge resection rather than classic regional resection is advised.

## Abbreviations

CEA: Carcinoembryonic antigen
CT: Computed tomography
EUS: Endorectal ultrasound
FDG-PET: Fluorodeoxyglucose positron emission tomography
GFAP: Glial fibrillary acidic protein
GI: Gastrointestinal
GIST: Gastrointestinal stromal tumor
NET: Neuroendocrine tumor
